# Efficacy and Safety of Atezolizumab Plus Bevacizumab in Patients With Advanced NSCLC Who Received Pretreatment With EGFR‐TKIs (ML41256): A Multicenter, Prospective, Single‐Arm, Phase 2 Trial

**DOI:** 10.1002/cam4.71469

**Published:** 2025-12-13

**Authors:** Wenfeng Fang, Jian Fang, Panwen Tian, Yun Fan, Qitao Yu, Xiaochun Zhang, Zhehai Wang, Xiaoyuan Liu, Yanjun Shi, Li Zhang

**Affiliations:** ^1^ Department of Medical Oncology Sun Yat‐sen University Cancer Center, State Key Laboratory of Oncology in South China, Collaborative Innovation Center for Cancer Medicine Guangzhou China; ^2^ Second Department of Thoracic Oncology Beijing Cancer Hospital Beijing China; ^3^ Department of Pulmonary and Critical Care Medicine, State Key Laboratory of Respiratory Health and Multimorbidity, Precision Medicine Key Laboratory of Sichuan Province, West China Hospital Sichuan University Chengdu China; ^4^ Lung Cancer Center/Lung Cancer Institute, West China Hospital Sichuan University Chengdu Sichuan China; ^5^ Department of Medical Oncology Zhejiang Cancer Hospital Hangzhou China; ^6^ Department of Respiratory Oncology Guangxi Medical University Affiliated Tumor Hospital Nanning China; ^7^ Cancer Precision Medical Center, The Affiliated Hospital of Qingdao University Qingdao China; ^8^ Respiratory Medical Oncology Ward II Shandong Cancer Hospital Jinan China; ^9^ Medical Affairs Shanghai Roche Pharmaceuticals Ltd. Shanghai China

**Keywords:** chemotherapy‐free, combination, EGFR‐TKI resistance, immunotherapy, non‐squamous NSCLC

## Abstract

**Background:**

Few treatment options are available for patients with epidermal growth factor receptor (EGFR) mutation‐positive metastatic non‐squamous non‐small cell lung cancer (NSCLC) who failed treatment with EGFR‐tyrosine kinase inhibitors (EGFR‐TKIs). We aimed to assess the efficacy and safety of atezolizumab plus bevacizumab in these patients.

**Methods:**

We conducted a single‐arm, Simon's minimax two‐stage adapted phase II study. Patients received atezolizumab 1200 mg plus bevacizumab 15 mg/kg once every 3 weeks. The primary endpoint was the objective response rate (ORR). Secondary endpoints included duration of response (DOR), time to response (TTR), progression‐free survival (PFS), overall survival (OS), and safety.

**Results:**

Between 14 August 2020 and 18 January 2021, 23 patients from seven sites in China were enrolled; all received study treatment. Twenty‐two patients were evaluable for ORR. At data cut‐off, median follow‐up was 16.4 months. The confirmed ORR was 18.2% (4/22) per RECIST v1.1. The number of responders did not cross the predefined threshold (> 6 patients) for stage II enrollment. For the four responding patients, the median TTR and DOR were 1.4 and 6.4 months, respectively. Median PFS and OS were 2.8 and 14.1 months, respectively. Atezolizumab plus bevacizumab had acceptable tolerability without any new safety signals identified. All patients experienced at least one treatment‐emergent adverse event (TEAE); four patients experienced serious TEAEs and one patient died of an unknown cause.

**Conclusions:**

The chemotherapy‐free atezolizumab plus bevacizumab regimen had limited efficacy but an acceptable safety profile in this exploratory study. Although current data suggest that chemotherapy may still be important for patients who failed EGFR‐TKIs, more treatment regimens with lower toxicity and higher efficacy for these patients, such as antibody–drug conjugates and bispecific antibodies, need to be explored in the future.

**Trial Registration:**

ClinicalTrials.gov identifier: NCT04426825

**Abbreviations**
ABCP: atezolizumab plus bevacizumab plus carboplatin/paclitaxel doublet chemotherapy
ALK: anaplastic lymphoma kinase
CI: confidence interval
CR: complete response
DCR: disease control rate
DOR: duration of response
ECOG: Eastern Cooperative Oncology Group
EGFR: epidermal growth factor receptor
FAS: full analysis set
IC: tumor‐infiltrating immune cells
imTEAE: immune‐mediated TEAE
NE: not evaluable
NSCLC: non‐small cell lung cancer
ORR: objective response rate
OS: overall survival
PD‐1: programmed cell death‐1
PD‐L1: programmed death ligand‐1
PFS: progression‐free survival
PR: partial response
Q3W: every 3 weeks
RECIST: Response Evaluation Criteria in Solid Tumors
TC: tumor cells
TEAE: treatment‐emergent adverse event
TKI: tyrosine kinase inhibitor
TTR: time to response

## 1 Introduction

Globally, lung cancer is the leading cause of cancer‐related death, accounting for 18% of all such deaths in 2020 [1]. For patients with non‐small cell lung cancer (NSCLC), testing for genetic alterations with prognostic and/or predictive significance is recommended prior to treatment selection [2]; such alterations include mutations in the epidermal growth factor receptor (*EGFR*) gene and rearrangement in the anaplastic lymphoma kinase (*ALK*) gene, among others [3]. Roughly one in three patients (32.3%) with NSCLC have *EGFR* mutations, with the highest prevalence reported among Asian patients (38.4%) and the lowest among Europeans (14.1%) [4]. In the past 20 years, several EGFR‐tyrosine kinase inhibitors (TKIs) have been approved for the treatment of advanced NSCLC with common *EGFR*‐sensitizing mutations [5]. Nevertheless, most patients eventually develop resistance to EGFR‐TKIs, regardless of the initial response [6].

The ideal treatment regimen after failure of EGFR‐TKIs remains unclear. Some patients, particularly those with asymptomatic disease or limited progression, may continue to obtain clinical benefit from EGFR‐TKI continuation (treatment beyond radiographic progression) [7]. For patients with advanced or metastatic EGFR‐mutant NSCLC, immunotherapy alone has limited benefit [8]; thus, second‐ or later‐line salvage treatments for patients with multiple lesions are usually composed of combination regimens. These may include platinum‐based doublet chemotherapy, chemotherapy plus either a programmed cell death‐1 (PD‐1) or programmed death ligand‐1 (PD‐L1) inhibitor, or chemotherapy plus PD‐1/PD‐L1 plus an antiangiogenic agent [9]. However, until recently, there has been a lack of data evaluating the relative efficacy of combination regimens in previously treated patients with NSCLC and *EGFR* mutations, resulting in an unmet clinical need for this specific subpopulation [7].

Recently, multiple studies have reported promising results for various combination regimens in second‐line treatment for patients with *EGFR* mutation‐positive NSCLC [10, 11, 12, 13]. Notably, encouraging clinical data from the IMpower150 study demonstrated the efficacy of atezolizumab plus bevacizumab plus carboplatin/paclitaxel doublet chemotherapy (ABCP) in an all‐comer chemotherapy‐naïve patient population with metastatic non‐squamous NSCLC, including those who had previously failed EGFR‐TKIs [14]. Overall, in patients who received ABCP every 3 weeks (Q3W) for four or six cycles, followed by maintenance therapy with atezolizumab, bevacizumab, or both, the objective response rate (ORR) was 63.5%, and the median progression‐free survival (PFS) was 8.3 months [14]; in the final analysis, the overall survival (OS) was reported to be 19.5 months [15]. In a subgroup analysis of patients with *EGFR*‐sensitizing mutations who had previously received TKIs, the median OS with ABCP was 27.8 months [16]. However, among patients with *EGFR* mutations, 66.7% reported Grade 3 or 4 treatment‐related adverse events (TEAEs).

One possible option to reduce treatment toxicity while retaining efficacy is to limit the number of therapeutic agents administered. The combination of atezolizumab plus bevacizumab (without additional doublet chemotherapy) was assessed in the TELMA study and was shown to be effective in the first‐line setting for patients with metastatic non‐squamous NSCLC without *EGFR* or *ALK* genomic alterations [17]. However, although a chemotherapy‐free regimen may provide an additional first‐line treatment choice for selected patients, the use of this regimen in patients who have tested positive for EGFR mutations yet subsequently failed EGFR‐TKI therapy has not yet been studied. Thus, the objectives of our study were to evaluate the efficacy and safety of atezolizumab plus bevacizumab in patients with EGFR mutation‐positive, stage IIIB–IV, non‐squamous NSCLC who had been pretreated with EGFR‐TKIs.

## 2 Materials and Methods

### 2.1 Patients

Patients who met the following eligibility criteria were included in the study: age ≥ 18 years; histologically or cytologically confirmed stage IIIB–IV non‐squamous NSCLC (per the Union Internationale contre le Cancer/American Joint Committee on Cancer staging system, 8th edition); measurable disease per Response Evaluation Criteria in Solid Tumors (RECIST) version 1.1; PD‐L1 expression ≥ 1% (by measurement of invasive tumor cells or tumor‐infiltrating immune cells); Eastern Cooperative Oncology Group (ECOG) performance status of 0 or 1; life expectancy ≥ 10 months; adequate hematologic, cardiac, and other organ function; and negative HIV, hepatitis B surface antigen or core antibody, and hepatitis C antibody tests. Furthermore, the following patients were eligible: those with a sensitizing mutation in the *EGFR* gene with disease progression/intolerance to treatment with at least one EGFR‐TKI; those with progression/intolerance to first‐line osimertinib or other third‐generation EGFR‐TKIs; those with progression/intolerance to first‐ or second‐generation EGFR‐TKIs and without evidence of a T790M mutation in the *EGFR* gene; and those with progression/intolerance to first‐ or second‐generation EGFR‐TKIs with evidence of the T790M mutation, but also progression/intolerance to osimertinib.

The key exclusion criterion was no prior chemotherapy or other systemic therapy for stage IIIB/IV disease. Patients with central nervous system metastases that were symptomatic, untreated, or actively progressing; those with a history of leptomeningeal disease; those with uncontrolled tumor‐related pain, pleural effusion, pericardial effusion, ascites, or hypercalcemia; those with the presence or history of autoimmune disease or immune deficiency, or who had undergone previous allogeneic stem cell or solid organ transplantation were also excluded. In addition, patients with any other condition, physical examination finding, or clinical laboratory finding that would contraindicate the use of the investigational drugs, could affect the interpretation of the results, or could render the patient at high risk of treatment complications were ineligible.

Written informed consent was provided by all patients or a suitable authorized representative prior to participation in the study.

### 2.2 Study Design and Treatment

This was an open‐label, single‐arm, phase II, multicenter study that assessed the efficacy/safety of atezolizumab plus bevacizumab (i.e., a chemotherapy‐free regimen) in patients with non‐squamous NSCLC after treatment failure of EGFR‐TKIs. Patients were enrolled at seven sites in China between August 14, 2020 and January 18, 2021.

The study included a screening period (day −28 to day −1), a treatment period, a treatment discontinuation visit period (≤ 30 days after the last dose), and a follow‐up period. All participants received atezolizumab 1200 mg plus bevacizumab 15 mg/kg, each administered by intravenous infusion Q3W. The first administration was atezolizumab, then followed by bevacizumab, and there was at least a 5‐min gap between dosing. No dose modifications were permitted for atezolizumab. We used Simon's minimax 2‐stage design (Figure S1).

### 2.3 Objectives and Assessments

The primary efficacy objective was the ORR. We defined this as the proportion of patients who had a complete response (CR) or partial response (PR) two consecutive times at least 4 weeks apart, which was determined by the study investigator according to the RECIST v1.1 guidelines. The secondary efficacy objectives were the duration of response (DOR), time to response (TTR), disease control rate (DCR), PFS, and OS. Patients were also assessed for PD‐L1 expression, EGFR‐activating mutations Ex19del and L858R, and the T790M mutation at baseline. PD‐L1 expression was analyzed using either the SP263 or SP142 assays, and “high expression” was defined as tumor cells (TC) ≥ 50% or tumor‐infiltrating immune cells (IC) ≥ 10% for the SP142 assay or TC ≥ 50% for the SP263 assay. The PD‐L1 ≥ 1% threshold was selected based on its established role as a biomarker to enrich for patients likely to respond to immunotherapy while maintaining broad eligibility.

Safety objectives included incidence/severity of adverse events, with severity determined according to the National Cancer Institute Common Terminology Criteria for Adverse Events version 5.0, and the incidence of serious and non‐serious immune‐mediated (im) TEAEs related to atezolizumab.

Tumor assessments were performed at baseline and every 6 weeks (±7 days) for the first 36 weeks following cycle 1, day 1; subsequent assessments occurred every 9 weeks (±7 days). We continued to perform assessments until patients had radiographic disease progression according to RECIST v1.1 or there was a loss of clinical benefit (for atezolizumab‐treated patients who continued treatment after radiographic disease progression); the study was terminated by the sponsor; or patients withdrew consent, discontinued the study, or died. Safety was assessed via evaluation of exposure to study treatment; TEAEs (categorized using the Medical Dictionary for Regulatory Activities version 25.1); and changes in laboratory test results, vital signs, and electrocardiography.

### 2.4 Statistical Analysis

The sample size was calculated based on the primary endpoint ORR. Simon's minimax two‐stage design was utilized to test the null hypothesis of an ORR ≤ 30% versus the alternative hypothesis of an ORR ≥ 50% at a type 1 error of 0.05 and power of 80%.

Based on this, we planned to include 19 evaluable patients at stage I of the study; if six or fewer patients responded to the treatment, enrollment would be stopped. Otherwise, an additional 20 patients would be evaluated (stage II). If > 16 patients in total responded to treatment (stage I and II combined), then it would be concluded that treatment resulted in an improved response (i.e., clinical benefit). Considering a drop‐out rate of 10%, a maximum of 44 patients were planned for enrolment.

The primary analysis of ORR was evaluated in the evaluable population, which we defined as all enrolled patients who received treatment and had data for at least one efficacy measurement (not including baseline data). Additionally, a supplementary analysis was conducted to assess ORR in the full analysis set (FAS); this included all enrolled patients who received the study treatment in any amount or at any dose. The safety analysis was performed in the safety analysis set, which we defined to include all enrolled patients who received any amount of study treatment.

Descriptive statistics, including the number, mean, standard deviation, median, and range, were used for continuous variables. Categorical variables were described as number (percent) in each category. For the endpoints of response, the number (percent) of responders with corresponding Clopper–Pearson 95% confidence intervals (CIs) were reported. For time‐to‐event endpoints, such as PFS and OS, the Kaplan–Meier method was used to produce Kaplan–Meier curves and to estimate the medians. The Brookmeyer–Crowley method was used to determine the 95% CI for the median. Survival probabilities at specific time points were also estimated using the Kaplan–Meier method; 95% CIs were determined using the Greenwood estimator. Missing data were not imputed. We performed all of the statistical analyses using SAS software version 9.4 (SAS Institute Inc., Cary, NC, USA).

## 3 Results

### 3.1 Patient Characteristics

**FIGURE 1:**
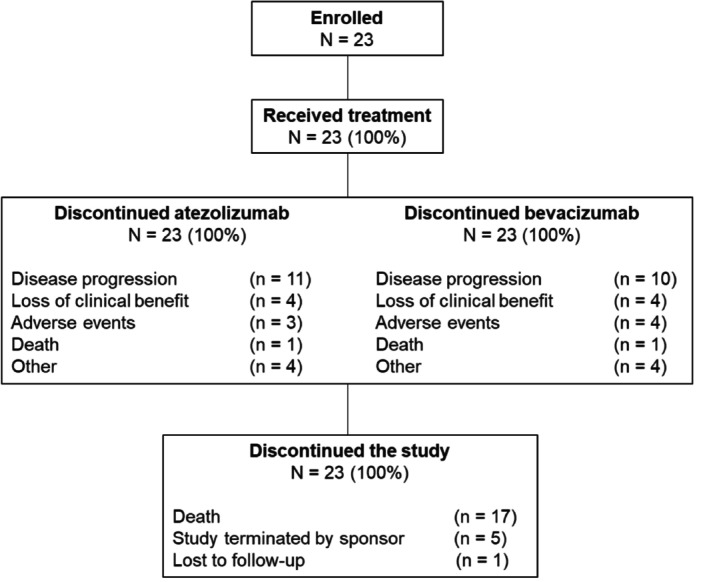
Patient disposition, including reasons for treatment and study discontinuation.

Between August 14, 2020 and January 18, 2021, 23 patients were enrolled at seven sites in China; all received at least one dose of study treatment. Twenty‐two patients were included in the evaluable population as one patient did not have post‐baseline efficacy measurements. At data cut‐off (February 11, 2023), 23 (100%) patients had discontinued study treatment (Figure 1). The most common reason for discontinuation of atezolizumab or bevacizumab treatment was disease progression, which was reported in 47.8% and 43.5% of patients, respectively. All patients had discontinued from the study, with the main reason being death.

The demographics and baseline characteristics of patients are provided in Table 1. Slightly more females (13/23; 56.5%) than males (10/23; 43.5%) were enrolled. The median patient age was 63.0 years (range, 37–76), and the majority of patients had non‐squamous adenocarcinoma (22/23; 95.7%) and an ECOG performance status of 1 (18/23; 78.3%). All patients had L858R mutation or exon 19 deletion in the *EGFR* gene; 5/23 (21.7%) and 10/23 (43.5%) patients, respectively, had brain or bone metastases.

**TABLE 1:** Patient demographic and baseline characteristics (full analysis set).

Characteristic	Study patients
	(*N* = 23)
Age (years)	
Median (range)	63.0 (37–76)
< 65 years, *n* (%)	14 (60.9)
≥ 65 years, *n* (%)	9 (39.1)
Sex (female/male), *n* (%)	13 (56.5)/10 (43.5)
Smoking history, *n* (%)	
Never	16 (69.6)
Previous	7 (30.4)
Current	0
ECOG performance status, *n* (%)	
0	5 (21.7)
1	18 (78.3)
Histologic type, *n* (%)	
Non‐squamous	23 (100)
Adenocarcinoma	22 (95.7)
NSCLC/NOS	1 (4.3)
Current stage, *n* (%)	
IV	23 (100)
PD‐L1 expression, *n* (%)	
Positive	23 (100)
High	6 (26.1)
*EGFR* mutation, *n* (%)	
L858R	11 (47.8)
Exon 19 deletion	12 (52.2)
Other	0
Brain metastasis, *n* (%)	
Yes	5 (21.7)
No	18 (78.3)
Bone metastasis, *n* (%)	
Yes	10 (43.5)
No	7 (30.4)
Missing	6 (26.1)

Abbreviations: ECOG, Eastern Cooperative Oncology Group; EGFR, epidermal growth factor receptor; LVEF, left ventricular ejection fraction; NOS, not otherwise specified; NSCLC, non‐small cell lung cancer; PD‐L1, programmed death ligand‐1.

### 3.2 Efficacy of Treatment With Atezolizumab Plus Bevacizumab

The median follow‐up time at the cut‐off was 16.4 months (range, 1.6–29.1 months). In the evaluable population assessed by the study investigator to calculate the primary efficacy outcome, no patients achieved a CR and four patients achieved a PR, resulting in a confirmed ORR of 18.2% (4/22; 95% CI, 5.2%–40.3%). Half of the evaluable patients achieved stable disease (11/22; 50.0%).

Efficacy outcomes in the efficacy evaluable population and the FAS are summarized in Table 2. The overall DCR in the FAS was 65.2% (95% CI, 42.7%–83.6%). A breakdown of ORR according to baseline factors such as the presence of brain metastases, *EGFR* L858R mutation, and PD‐L1‐high expression is shown in Table S1.

**TABLE 2:** Summary of tumor response.

	Efficacy evaluable population
	(*N* = 22)
Primary efficacy outcome ORR, % (95% CI)	18.2% (5.2–40.3)
BOR, *n* (%)	
CR	0
PR	4 (18.2)
SD	11 (50.0)
PD	7 (31.8)

	Full analysis set
	(*N* = 23)
ORR, % (95% CI)	17.4 (5.0–38.8)
DCR, % (95% CI)	65.2 (42.7–83.6)
Median TTR^a^, months (range)	1.4 (1.2–2.3)
Median DOR^a^, months (95% CI)	6.4 (2.7–NE)
DOR rate^a^, % (95% CI)	
At 6 months	50.0 (5.8–84.5)
At 9 months	25.0 (0.9–66.5)
Median PFS, months (95% CI)	2.8 (1.4–4.1)
PFS rate, % (95% CI)	
At 6 months	13.9 (3.5–31.4)
At 12 months	4.6 (0.3–19.3)
Median OS, months (95% CI)	14.1 (8.8–22.1)
OS rate, % (95% CI)	
At 12 months	52.2 (30.5–70.0)
At 24 months	30.4 (13.5–49.3)

Abbreviations: BOR, best overall response; CI, confidence interval; CR, complete response; DCR, disease control rate; DOR, duration of response; NE, not evaluable; ORR, objective response rate; OS, overall survival; PD, progressive disease; PFS, progression‐free survival; PR, partial response; SD, stable disease; TTR, time to response.

^a^ In patients who achieved a BOR of CR or PR (*n* = 4).

The number of responders did not cross the predefined threshold (> 6 patients) for stage II enrollment. Thus, stage II was not formally initiated. For the four responding patients, the median TTR was 1.4 months (range, 1.2–2.3), and the median DOR was 6.4 months (95% CI, 2.7–not evaluable [NE]).

**FIGURE 2:**
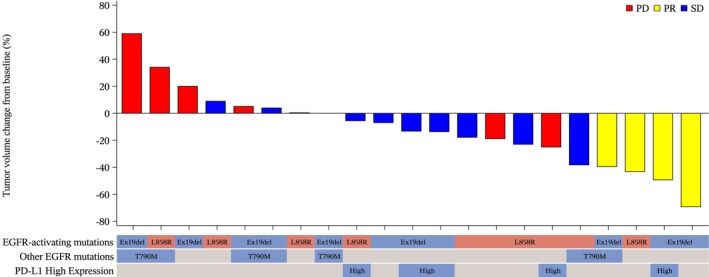
Waterfall plot of the best percentage change of the patient's SLD from baseline (full analysis set). One patient who was not evaluable for target lesion assessment and one patient with no post‐baseline target lesion assessment were excluded. PD, progressive disease; PR, partial response; SD, stable disease; SLD, sum of target lesion diameters.

The maximum change from baseline in the sum of diameters for the target lesions is shown in Figure 2; patients who achieved a PR also had the largest decrease in tumor volume during the course of treatment. Out of the four patients with a PR, three had the exon 19 deletion (ex19del) mutation, and one had a combination of ex19del and T790M mutations. Additionally, one patient had high expression of PD‐L1. Because of the limited number of patients, the biomarker analysis did not reveal any trends in the presence of specific *EGFR* mutations or PD‐L1 expression with changes in tumor volume (Figure 2).

**FIGURE 3:**
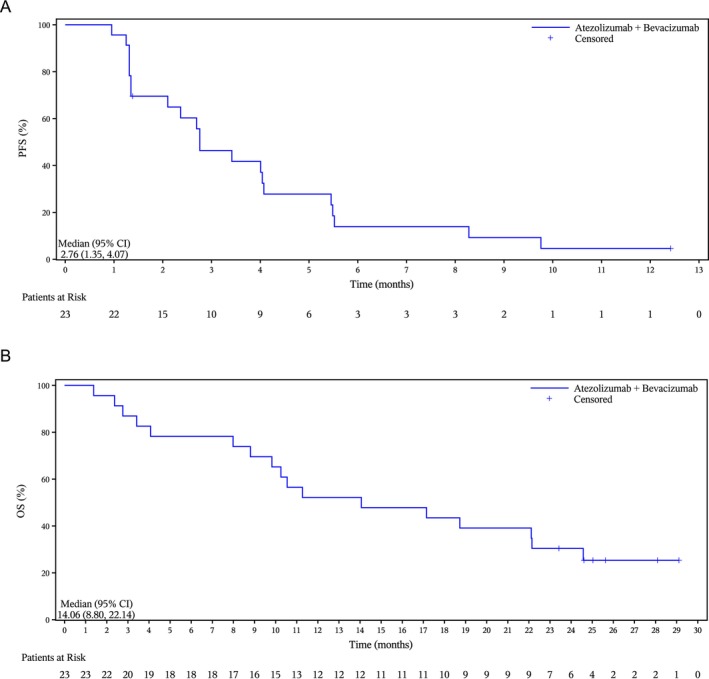
Kaplan–Meier survival analyses showing (A) RECIST‐assessed PFS and (B) OS (full analysis set). CI, confidence interval; NE, not evaluable; OS, overall survival; PFS, progression‐free survival; RECIST, Response Evaluation Criteria in Solid Tumors version 1.1.

At the data cut‐off, 21/23 (91.3%) patients had disease progression or had died. The median PFS was 2.8 months (95% CI, 1.4–4.1; Table 2 and Figure 3A); 17/23 (73.9%) patients had died and the median OS was 14.1 months (95% CI, 8.8–22.1; Table 2 and Figure 3B).

### 3.3 Safety Outcomes

Safety data are summarized in Table 3. Overall, no new safety signals were observed. All 23 patients available for the safety evaluation experienced at least one TEAE. One patient experienced a TEAE leading to death/study discontinuation, six patients experienced a TEAE leading to drug interruption, and four patients experienced a TEAE leading to drug withdrawal.

**TABLE 3:** Summary of safety results (safety population).

TEAE, *n* (%)	Study patients
	(*N* = 23)
Any TEAE	23 (100)
Related to atezolizumab	23 (100)
Related to bevacizumab	20 (87.0)
Any serious TEAE	4 (17.4)
Related to atezolizumab	4 (17.4)
Related to bevacizumab	2 (8.7)
Any TEAE leading to study discontinuation	1 (4.3)
Related to atezolizumab	1 (4.3)
Related to bevacizumab	1 (4.3)
Any TEAE leading to study drug interruption	6 (26.1)
Related to atezolizumab	4 (17.4)
Related to bevacizumab	4 (17.4)
Any TEAE leading to study drug withdrawal	4 (17.4)
Related to atezolizumab	3 (13.0)
Related to bevacizumab	2 (8.7)
Any TEAE leading to death	1 (4.3)
Related to atezolizumab	1 (4.3)
Related to bevacizumab	1 (4.3)
TEAE of special interest	16 (69.6)
Related to atezolizumab	15 (65.2)
Related to bevacizumab	12 (52.2)

TEAEs occurring in ≥ 10% of the study population	Any grade	Grades 3–5
Proteinuria	7 (30.4)	1 (4.3)
Anemia	5 (21.7)	0
Decreased appetite	5 (21.7)	0
Fatigue	6 (26.1)	0
Hypercholesterolemia	5 (21.7)	0
Hypoalbuminemia	5 (21.7)	0
Hypertension	6 (26.1)	2 (8.7)
Platelet count decreased	5 (21.7)	1 (4.3)
ALT increased	4 (17.4)	0
AST increased	6 (26.1)	0
Blood TSH increased	5 (21.7)	0
Upper respiratory tract infection	4 (17.4)	0
Hyperglycemia	3 (13.0)	0
Hypertriglyceridemia	3 (13.0)	0
Hypothyroidism	4 (17.4)	0
Pruritus	3 (13.0)	0
Urinary tract infection	5 (21.7)	0
Constipation	3 (13.0)	0
Diarrhea	3 (13.0)	0
Muscular weakness	3 (13.0)	0
Pain in extremity	5 (21.7)	1 (4.3)
Cough	3 (13.0)	0
Weight decreased	5 (21.7)	1 (4.3)

Abbreviations: ALT, alanine aminotransferase; AST, aspartate aminotransferase; TEAE, treatment‐emergent adverse event; TSH, thyroid‐stimulating hormone.

The most common TEAEs were proteinuria (*n* = 7) and fatigue, hypertension, and increased aspartate aminotransferase (*n* = 6 each). The most common Grade ≥ 3 TEAE was hypertension (*n* = 2). Protocol‐mandated measures ensured that these known toxicities were proactively monitored and managed according to pre‐specified criteria to ensure patient safety. Four patients experienced seven serious TEAEs (classified by Preferred Term) of malaise (*n* = 1), gastroenteritis (*n* = 1), pneumonia (*n* = 1), upper respiratory tract infection (*n* = 1), platelet count decreased (*n* = 1), left ventricular dysfunction (*n* = 1), and death (*n* = 1). One patient experienced a Grade 5 TEAE of death with an unknown cause, which was judged by the investigator to have an unknown relationship with the study treatments due to a lack of medical information and was eventually classified as related to atezolizumab and bevacizumab treatment. TEAEs of special interest occurred in 16 (69.6%) patients, which were judged (in the opinion of the investigator) to be related to atezolizumab in 15/23 patients (65.2%) and included imTEAEs and infusion‐related reactions. The imTEAEs were hepatitis (*n* = 8; 34.8%), hypothyroidism (*n* = 9; 39.1%), and severe cutaneous reactions (*n* = 2; 8.7%); all were Grade 1. One patient (4.3%) experienced an infusion‐related reaction, which was Grade 3.

## 4 Discussion

There are limited treatment options for patients with EGFR‐sensitizing mutation‐positive (ex19del/L858R) NSCLC who have failed EGFR‐TKIs. Chemotherapy or bevacizumab plus chemotherapy is currently the accepted standard of care in China, but the outcome is unsatisfactory with a PFS of 4–7 months [13, 16, 18]. This highlights the urgent need for new treatment options. Immunotherapy combining anti‐angiogenic agents plus chemotherapy has shown an OS benefit in this subpopulation, with respective OS and PFS of 19.2 and 8.3 months [16]. As such, the ABCP regimen may be considered a treatment option for patients with EGFR‐TKI failure in Europe [19]. However, toxicity may be an issue for some patients, with high rates of Grade ≥ 3 TEAEs in some subpopulations [16]. At the time our study was initiated (August 2020), there were limited published trial data for chemotherapy‐free regimens combining immunotherapy with anti‐angiogenic therapy specifically for EGFR TKI‐resistant NSCLC. This prompted us to explore a chemotherapy‐free regimen of atezolizumab plus bevacizumab, aiming to provide an additional, and potentially less toxic, option for previously treated patients with EGFR mutation‐positive NSCLC. Another notably innovative component of our trial was the targeted enrollment of patients with PD‐L1‐positive tumors. Considering that PD‐L1 expression is an established biomarker with the potential to guide immunotherapy, our approach aimed to more accurately identify a patient subgroup likely to benefit from this chemotherapy‐free strategy.

However, the primary analysis of the ORR was 18.2% in the ORR‐evaluable population in this open‐label, single‐arm, phase II, multicenter study. The number of responders did not cross the predefined threshold for stage II enrollment, and the study was therefore stopped for futility. This chemotherapy‐free regimen showed limited efficacy but manageable safety. Despite the early termination of our study, some responses were observed in certain patients. We detected the PD‐L1 expression and EGFR mutation types in these patients and attempted to identify specific subgroups that could benefit from this chemotherapy‐free regimen, but because of the limited sample size, we could not definitively identify which subpopulations might benefit most from this regimen. In addition, an exploratory stratified analysis based on the presence of adverse prognostic indicators such as brain metastases and *EGFR* L858R mutation found that patients in these subgroups had less favorable outcomes compared with the overall population. This suggests that the atezolizumab plus bevacizumab chemotherapy‐free regimen did not provide any specific benefit for these patient subgroups, although the small sample sizes preclude any definitive conclusions. Resistance in the EGFR‐TKI treatment failure setting involves complex mechanisms, including immunosuppressive factors that are not yet fully understood [20]. Therefore, further research in this area is essential to the development of individualized treatment regimens.

There have been several other small prospective exploratory studies also examining whether the “chemo‐free” model of immune checkpoint inhibitors combined with anti‐angiogenic therapy can bring benefits to patients with resistance to EGFR‐TKIs. A phase II study evaluated camrelizumab plus apatinib, with a confirmed ORR of 18.6%, a median PFS of 2.8 months, and a median follow‐up period of 15.7 months [21]. Two other phase II studies evaluated TQB2450 (a PD‐L1 antibody) in combination with benmelstobart plus anlotinib [22] or anlotinib plus sintilimab [23] in patients who failed EGFR‐TKIs. The respective ORRs were 25.5% and 38.1%, with median PFS of 9.0 and 7.0 months [22, 23]. Considering that these were exploratory studies with small sample sizes and inconsistent results, clear conclusions cannot be drawn.

Recently, there has been much interest in exploring combination regimens in this area, and several recent studies have reported promising data using combination regimens containing chemotherapy in pretreated EGFR mutation‐positive patients [10, 11, 12, 13]. The phase III ORIENT‐31 study of sintilimab plus a bevacizumab biosimilar plus chemotherapy reported improved median PFS (6.9 months) compared with chemotherapy alone (4.3 months) [13]. The phase III ATTLAS study showed that the ABCP regimen significantly extended PFS to 8.48 months compared with 5.62 months with chemotherapy in EGFR‐ or ALK‐mutated NSCLC, while the OS remained similar (20.63 vs. 20.27 months) [24]. Additional phase II studies examining regimens with atezolizumab plus bevacizumab plus platinum‐based chemotherapy have also supported similar OS outcomes in patients with NSCLC carrying *EGFR* mutations who had previously received a TKI. For example, in the phase II NEJ043 study with ABCP, the median OS was 23.1 months [25] and in the phase II NCT04147351 trial that treated patients with atezolizumab and bevacizumab plus pemetrexed and carboplatin/cisplatin, the OS was found to be 27.6 months in patients with PD‐L1 ≥ 1% and 20.2 months in those with PD‐L1 < 1% [26]. Although comparisons between trials should be made cautiously, the differences in OS rates with these regimens containing chemotherapy appear to greatly exceed the OS observed in the current study (14.1 months) without chemotherapy. In the HARMONi‐A (AK112‐301) study, the PD‐1/VEGF bispecific antibody ivonescimab in combination with a chemotherapy regimen showed a significant improvement in PFS at a median follow‐up of 7.9 months. The median PFS in the ivonescimab combination chemotherapy group and the control group were 7.1 and 4.8 months, respectively, with a hazard ratio of 0.46 (*p* < 0.001) [27]. These results highlighted that immune checkpoint inhibitors combined with anti‐angiogenic drugs and chemotherapy can improve survival outcomes for patients with advanced NSCLC who have failed EGFR‐TKI treatment.

Studies of immune checkpoint inhibitors combined with chemotherapy, such as CheckMate 722 and KEYNOTE 789, have explored the efficacy of immune checkpoint inhibitors combined with chemotherapy in the treatment of patients who have failed EGFR‐TKI treatment. However, neither of these two combination regimens showed superior efficacy to chemotherapy [28, 29].

Other innovative combination treatment options are also being explored in this population. The global phase III trial MARIPOSA‐2 evaluated the combination of amivantamab (EGFR/MET bispecific monoclonal antibody) plus carboplatin–pemetrexed chemotherapy, with and without lazertinib, in patients with EGFR‐mutated advanced NSCLC after progression on osimertinib. Both amivantamab–chemotherapy and amivantamab–lazertinib–chemotherapy combinations significantly improved PFS with median PFS of 6.3 and 8.3 months, respectively, versus 4.2 months for chemotherapy alone [30]. In the phase II study HERTHENA‐Lung01, patritumab deruxtecan (HER3‐DXd), an antibody–drug conjugate (ADC) consisting of a monoclonal antibody targeting HER3 linked to a topoisomerase I inhibitor payload, demonstrated a confirmed ORR of 29.8% and a median OS of 11.9 months in patients with advanced EGFR‐mutated NSCLC previously treated with EGFR‐TKIs and platinum‐based chemotherapy [31]. The phase III randomized controlled trial HERTHENA‐Lung02 also demonstrated positive results [32]. In the open‐label, phase III TROPION‐Lung01 study, patients treated with datopotamab deruxtecan had longer median PFS than those treated with docetaxel (4.4 months vs. 3.7 months), primarily driven by patients with non‐squamous histology [33].

In light of these findings, chemotherapy continues to play an important role in treating patients with EGFR‐TKI treatment failure who harbor EGFR‐sensitizing mutations, and combination strategies and novel therapies with lower toxicity and higher efficacy need to be explored in the future.

The limitations of this study include the limited sample size and single‐arm design. Additionally, as the study was terminated early for futility, this restricted our ability to evaluate co‐mutations and other biomarker data.

## 5 Conclusion

This study demonstrated limited ORR benefits from a regimen of atezolizumab plus bevacizumab and an acceptable level of safety for patients with advanced non‐squamous NSCLC who had failed EGFR‐TKIs. While it is possible that chemotherapy‐containing regimens may overcome tumor resistance to enhance outcomes, novel approaches such as antibody–drug conjugates and bispecific antibodies hold promise for achieving higher efficacy with potentially lower toxicity. Future research should explore these innovative therapies to optimize treatment strategies for this patient population.

## Author Contributions

Wenfeng Fang: investigation, project administration, resources, writing – original draft, writing – review and editing. Jian Fang: investigation, resources, writing – review and editing. Panwen Tian: investigation, resources, writing – review and editing. Yun Fan: investigation, resources, writing – review and editing. Qitao Yu: investigation, resources, writing – review and editing. Xiaochun Zhang: investigation, resources, writing – review and editing. Zhehai Wang: investigation, resources, writing – review and editing. Xiaoyuan Liu: conceptualization, data curation, formal analysis, funding acquisition, project administration, supervision, writing – review and editing. Yanjun Shi: data curation, formal analysis, validation, writing – review and editing. Li Zhang: conceptualization, formal analysis, investigation, project administration, resources, supervision, writing – original draft, writing – review and editing.

## Funding

This study was funded by Shanghai Roche Pharmaceuticals Ltd.

## Ethics Statement

The study was conducted in full conformance with Good Clinical Practice guidelines, the principles of the Declaration of Helsinki, and all applicable national laws and regulations. All study documentation was approved by the relevant Review Boards or Ethics Committees (Leading site: No. A2020‐011‐01, Ethics Committee of Sun Yat‐sen University Cancer Center).

## Consent

All patients or their legally authorized representatives provided written informed consent for study participation.

## Conflicts of Interest

Wenfeng Fang, Jian Fang, Panwen Tian, Yun Fan, Qitao Yu, Xiaochun Zhang, Zhehai Wang, and Li Zhang received investigator fees from Shanghai Roche Pharmaceuticals Ltd. Xiaoyuan Liu and Yanjun Shi are employees of Shanghai Roche Pharmaceuticals Ltd.

## Supporting information


**Data S1:** cam471469‐sup‐0001‐Supinfo1.zip.

## Data Availability

For up to date details on Roche's Global Policy on the Sharing of Clinical Information and how to request access to related clinical study documents, see https://go.roche.com/data_sharing. Anonymized records for individual patients across more than one data source external to Roche cannot, and should not, be linked due to a potential increase in the risk of patient re‐identification.
