# Supplementary material for: Efficacy and Safety of Atezolizumab Plus Bevacizumab in Patients With Advanced NSCLC Who Received Pretreatment With EGFR‐TKIs (ML41256): A Multicenter, Prospective, Single‐Arm, Phase 2 Trial
Source: Cancer Med. 2025 Dec 13;14(24):e71469. doi: 10.1002/cam4.71469 (PMC12701562; doi:10.1002/cam4.71469)
Supplement: Supplementary file 1 — Data S1: cam471469‐sup‐0001‐Supinfo1.zip. [file CAM4-14-e71469-s001.zip › cam471469-sup-0001-FigureS1-S1@ML41256_Supp Fig 1.pptx]

## Slide 1
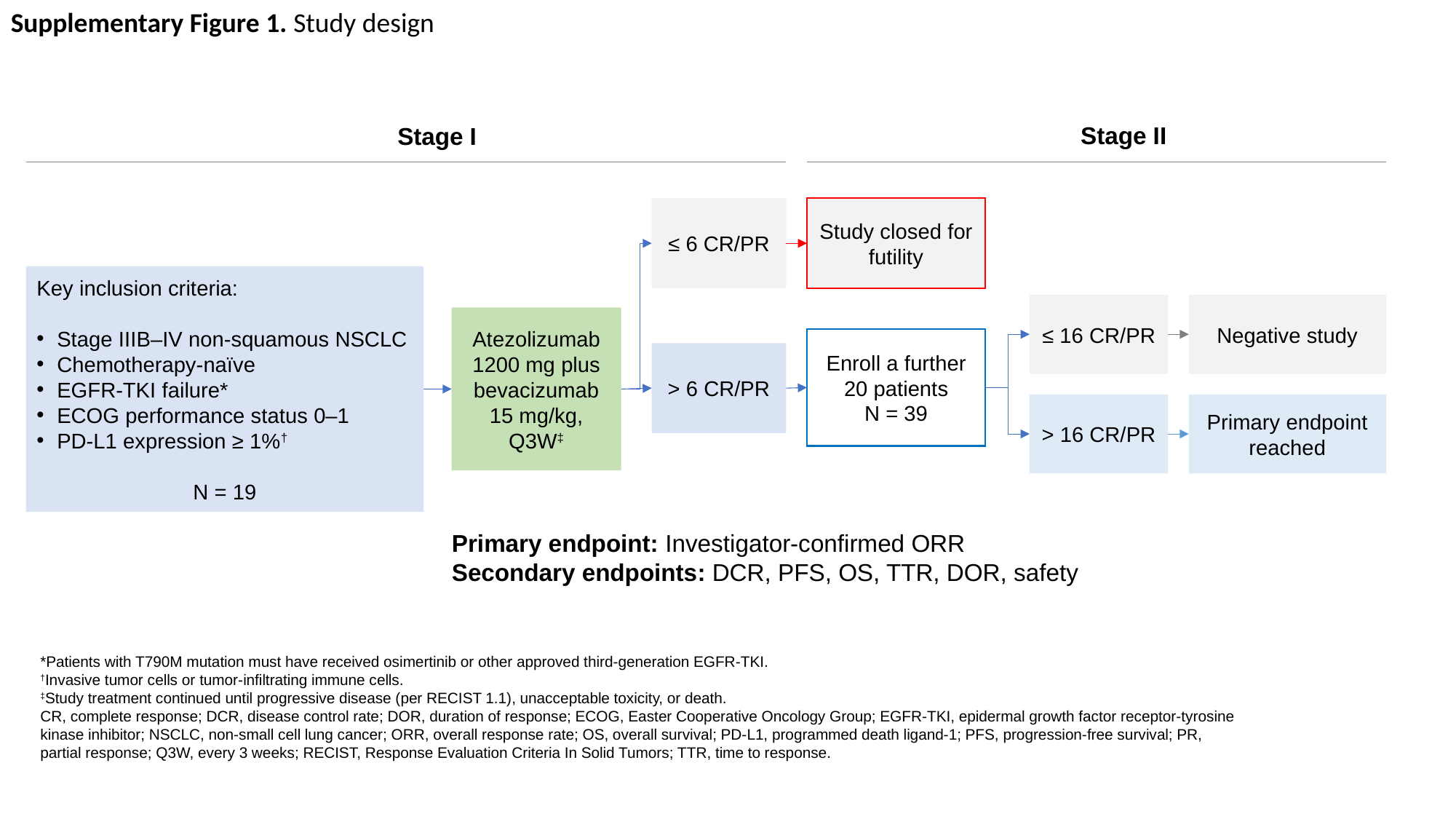

Supplementary Figure 1. Study design
Stage II
Stage I
≤ 6 CR/PR
Study closed for futility
Key inclusion criteria:
Stage IIIB–IV non-squamous NSCLC
Chemotherapy-naïve
EGFR-TKI failure*
ECOG performance status 0–1
PD-L1 expression ≥ 1%†
N = 19
≤ 16 CR/PR
Negative study
Atezolizumab
1200 mg plus
bevacizumab
15 mg/kg, Q3W‡
Enroll a further 20 patients
N = 39
> 6 CR/PR
> 16 CR/PR
Primary endpoint reached
Primary endpoint: Investigator-confirmed ORR
Secondary endpoints: DCR, PFS, OS, TTR, DOR, safety
*Patients with T790M mutation must have received osimertinib or other approved third-generation EGFR-TKI.
†Invasive tumor cells or tumor-infiltrating immune cells.
‡Study treatment continued until progressive disease (per RECIST 1.1), unacceptable toxicity, or death.
CR, complete response; DCR, disease control rate; DOR, duration of response; ECOG, Easter Cooperative Oncology Group; EGFR-TKI, epidermal growth factor receptor-tyrosine kinase inhibitor; NSCLC, non-small cell lung cancer; ORR, overall response rate; OS, overall survival; PD-L1, programmed death ligand-1; PFS, progression-free survival; PR, partial response; Q3W, every 3 weeks; RECIST, Response Evaluation Criteria In Solid Tumors; TTR, time to response.
