# Supplementary material for: Efficacy and Safety of Atezolizumab Plus Bevacizumab in Patients With Advanced NSCLC Who Received Pretreatment With EGFR‐TKIs (ML41256): A Multicenter, Prospective, Single‐Arm, Phase 2 Trial
Source: Cancer Med. 2025 Dec 13;14(24):e71469. doi: 10.1002/cam4.71469 (PMC12701562; doi:10.1002/cam4.71469)
Supplement: Supplementary file 1 — Data S1: cam471469‐sup‐0001‐Supinfo1.zip. [file CAM4-14-e71469-s001.zip › cam471469-sup-0002-Supinfo1@ML41256_Supplementary Information.docx]

**Supplemental Information**

**Supplemental Figure 1.** Study design

*Patients with T790M mutation must have received osimertinib or other approved third-generation EGFR-TKI.

^†^Invasive tumor cells or tumor-infiltrating immune cells.

^‡^Study treatment continued until progressive disease (per RECIST 1.1), unacceptable toxicity, or death.

CR, complete response; DCR, disease control rate; DOR, duration of response; ECOG, Easter Cooperative Oncology Group; EGFR-TKI, epidermal growth factor receptor-tyrosine kinase inhibitor; NSCLC, non-small cell lung cancer; ORR, overall response rate; OS, overall survival; PD-L1, programmed death ligand-1; PFS, progression-free survival; PR, partial response; Q3W, every 3 weeks; RECIST, Response Evaluation Criteria In Solid Tumors; TTR, time to response.

**Supplemental Table 1.** Summary of tumor response according to select baseline characteristics

|  | **Brain metastases**  **(n = 5)** | ***EGFR* L858R mutation**  **(n = 11)** | **PD-L1-high**  **(n = 6)** |
| --- | --- | --- | --- |
| BOR, n (%)* |  |  |  |
| CR | 0 | 0 | 0 |
| PR | 0 | 1 (9.1) | 1 (16.7) |
| SD | 3 (60.0) | 6 (54.5) | 3 (50.0) |
| PD | 2 (40.0) | 4 (36.4) | 2 (33.3) |
| ORR, % (95% CI) | 0 (NE–NE) | 9.1 (0.2–41.3) | 16.7 (0.4–64.1) |

BOR, best overall response; CI, confidence interval; CR, complete response; NE, not evaluable; ORR, objective response rate; PD, progressive disease; PR, partial response; SD, stable disease.
